# Fear of cancer recurrence and perceived pain in patients with breast cancer: A network analysis approach

**DOI:** 10.1016/j.apjon.2025.100763

**Published:** 2025-07-23

**Authors:** Furong Chen, Yiguo Deng, Siyu Li, Qihan Zhang, Zhirui Xiao, M. Tish Knobf, Zengjie Ye

**Affiliations:** a School of Nursing, Guangzhou Medical University, Guangzhou, Guangdong Province, China; b School of Nursing, University of South China, Hengyang, Hunan Province, China; c School of Nursing, Yale University, Orange, CT, United States

**Keywords:** Breast cancer, Fear of cancer recurrence, Pain catastrophizing, Network analysis

## Abstract

**Objective:**

This study aimed to identify core symptom nodes and examine directional relationships within the networks of fear of cancer recurrence (FCR) and pain catastrophizing (PC), and to investigate high-impact targets for intervention.

**Methods:**

From September to November 2024, a total of 346 eligible patients with breast cancer were enrolled from a multi-center trial named as Be Resilient to Breast Cancer (BRBC). The Fear of Cancer Recurrence Inventory and the Pain Catastrophizing Scale was used to collect data. A Gaussian network analysis was performed to identify the key components for FCR, PC and the connections between them. Bayesian networks were used to identify pathways of symptom activation at the symptom-level network architecture, and computer-simulated interventions were used to identify specific intervention targets.

**Results:**

In the analysis of separate networks, “Severity” emerged as the primary component of FCR, exhibiting the highest centrality metrics. For the PC, “Terrible” was identified as the central symptom, with notable centrality values. The dimension “Assurance” and the item “Awful” served as critical bridging elements, facilitating the interaction between FCR and PC when they co-occur. Bayesian network analysis identified 36 directed edges, with “Insight” in FCR and “Anxious” in PC acting as parent nodes, indicating their influential roles in the network structure. Computer-simulated interventions demonstrated that amplifying the “Terrible” node in PC maximized the total score and network connectivity. Conversely, attenuating the “Triggers” node in FCR minimized the total score.

**Conclusions:**

This study demonstrates that FCR and PC exhibit distinct network structures, which have their own specific core symptoms and corresponding core bridging nodes when the two coexist. This may serve as primary targets for personalized interventions for patients with breast cancer.

## Introduction

The incidence of breast cancer (BC) stands as the dominant cancer type among women globally, exhibiting increasing trends in both the number of cases and survival outcomes.^1^ Despite the significant progress in prolonging the life span of patients with BC,^2^ approximately 90% of deaths remain linked to the elevated recurrence of BC, perpetuating a significant challenge that remains to be fully addressed.^3^ Fear of cancer recurrence (FCR) is a prevalent psychological distress response among BC survivors,^4^ which can be persistent^5^ and adversely affect quality of life and psycho-physical health.^6^ Although numerous randomized controlled trials have investigated the effects of psychological interventions aimed at reducing FCR, the results have been inconclusive.^7^, ^8^, ^9^ The identification of its core symptoms may lead to important prerequisites for targeted therapeutic intervention.

Approximately 40%–90% of cancer patients experience pain, with over 30% reporting moderate to severe levels,^10^ resulting in frequent pessimism such as frustration and negativity.^11^ Pain Catastrophizing (PC) refers to rumination, exaggeration and helplessness of a realistically occurring or anticipated pain experience.^12^ Women who experienced persistent pain one year after surgery, compared to those without pain, seemed to have altered pain modulation processes, with catastrophic thinking about pain appearing to contribute to the persistence of pain.^13^ Greater catastrophic thinking about pain is associated with upper limb functional disabilities, quality of life and increased psychological distress.^14^^,^^15^ It is worth noting that few studies on PC intervention targets specifically for Chinese patients with BC. Painful symptoms in cancer patients can be particularly worrisome as they may signify and are often perceived as indicating worsening disease or recurrence.^16^^,^^17^

On the other hand, cancer survivors who report new or lingering pain symptoms also have increased levels of FCR.^18^ Pain intensity and PC have been highlighted as significant factors contributing to augmented FCR among survivors of childhood cancers,^19^ but further clarification is needed in BC. It has also been shown that there is no significant relationship exists between PC and FCR.^20^ This inconsistency may arise from divergent cultural contexts, the use of differing assessment methodologies, and other variables. Given these inconsistencies, a critical question arises: how do components of FCR and PC interact at the symptom level among patients with BC, and can network-based modeling clarify the nature of these associations?

Unique cultural, psychological, and health system-specific factors shape the experiences of FCR and PC in Chinese patients with BC. Culturally, traditional Chinese beliefs often associate cancer with inevitability or fate,^21^ which may amplify FCR, while stoicism toward pain can lead to underreporting of PC, complicating clinical management. Psychologically, stigma surrounding mental health and limited emotional expression may exacerbate FCR and hinder coping with chronic pain.^22^ From a health system perspective, disparities in access to psychological support and pain management resources in China,^23^ compared to Western settings, underscore the need for tailored interventions. These factors collectively highlight the importance of investigating FCR and PC in Chinese patients with BC to inform culturally sensitive and effective clinical strategies.

Previous research^24^^,^^25^ has predominantly relied on aggregate scores to report the intensity of psychological symptoms, overlooking the intricate relationship of specific psychological problems, resulting in failure to capture the nuanced connections between complex psychological phenomena.^26^^,^^27^ Network analysis is designed to identify factors that are interconnected.^28^ This study utilized a network analysis approach to investigate the associations of the components of FCR and PC among a cohort of female patients with BC. We hypothesize that: (1) FCR and PC each possess distinct core symptom nodes within their respective network structures; (2) specific symptoms serve as bridges between the two networks when FCR co-occurs with PC; and (3) network structures vary across patients at different tumor stages.

## Methods

### Participants

From September to November 2024, We enrolled 363 women with BC from the “Be Resilient to Breast Cancer (BRBC)” program for the survey.^29^, ^30^, ^31^, ^32^, ^33^ A comprehensive description of the inclusion and exclusion criteria has been provided in previous publications.^34^, ^35^, ^36^, ^37^ An initial data review showed that 17 questionnaires (4.7%) were invalid due to missing values. The study ultimately included 346 participants, yielding a 95.3% response rate.

### Sample size

According to the sample size requirement for network analysis, the sample size should be at least higher than the total number of parameters.^38^ In this study, a total of 20 nodes are to be constructed, thus the threshold parameter ​= ​20, the pairwise correlation parameter =(20∗19)/2 ​= ​190, and the minimum sample size is 210 cases. Anticipating a 20% attrition rate, the calculated necessary sample size was determined to be 263.

### Measurements

#### Demographic information

We collected the participants’ demographic information, including age, marital status, education, residence, medical insurance, occupation, household Income (RMB), cancer stage, fertility status, family history of malignancy, and menopause status building on previous research.^14^^,^^39^

#### Assessment of fear of cancer recurrence

We used the Fear of Cancer Recurrence Inventory (FCRI)^40^ to assess FCR, which was considered one of the most psychologically powerful measures.^41^ It consists of 42 items across 7 dimensions, covering trigger factors, severity, psychological distress, functioning impairment, insight, reassurance, and coping strategies. A 5-point Likert scale was employed (0–4 with 4 being higher), with item 13 requiring reverse scoring. The higher the total score, the higher the FCR. In this study, the Cronbach's *α* of this scale was 0.846.

#### Assessment of PC

The Pain Catastrophizing Scale (PCS)^42^ was used to assess the PC of patients with BC. The scale consists of 3 dimensions of rumination, exaggeration, and helplessness, totaling 13 items. Each item is rated on a 5-point Likert scale, ranging from 0 (“not at all”) to 4 (“all the time”), with the total score ranging from 0 to 52 points. In this study, the Cronbach's *α* was 0.813.

### Statistical analysis

Descriptive statistics were performed using IBM SPSS 28.0 software, and network analyses were performed with different R packages in the R 4.4.0 version.

Firstly, the Goldbricker package was employed to perform a topological overlap test, examining all possible combinations of correlations to detect item redundancy.^43^ All items were retained following the Goldbricker procedure, resulting in a final network comprising 20 nodes. The initial symptom network was constructed using a Gaussian graphical model (GGM) based on partial correlations, with polychoric correlations used for ordinal variables via the cor ​= ​“cor_auto” parameter, estimated with the Extended Bayesian Information Criterion graphical lasso (EBICglasso) method to identify robust associations between symptoms of FCR and PC. We then implemented bridge node analysis to assess bridge centrality across all network nodes, employing bridge strength as the measurement index.^44^ Node strength was defined as the sum of the absolute edge weights connected to a node, reflecting its overall direct connectivity. Betweenness refers to the extent to which a node lies on the shortest paths between other nodes, indicating its role as a connector. Closeness captures how close a node is to all other nodes in the network, based on average shortest path lengths.

In addition, we used a case-dropping subset bootstrap to calculate the correlation stability (CS) coefficient. Typically, a CS coefficient greater than 0.25 is required.^38^ To assess the accuracy of edge weights, we employed nonparametric bootstrapping procedures to calculate the 95% confidence intervals (CIs) for each edge value.^45^ We performed the Network Comparison Test (NCT) between patients with early BC (stages I and II) and patients with advanced BC (stages III and IV) in combined FCR and PC to detect differences between two networks.^46^

Thirdly, we performed Bayesian network analysis to infer potential causal directions among symptoms.^47^ Only edges that maintained consistent directionality in at least 75% of iterations were retained.^48^ The average size of the Markov blanket (MBS) and the branching factor (BF) can reflect the complexity of the dependence relationship between variables, with larger values of them indicating a more complex network structure and richer variable, dependence.^49^

Lastly, under an Ising-model framework, we used NodeIdentifyR for computer-simulated interventions. Variables were dichotomized (> 0). The “simulateResponses” function perturbed each node by ± 2 SD. R^2^ values from mgm model measured prediction accuracy. Edge weights and thresholds from Ising network were used, with GS scores summed and visualized via ggplot2. For each symptom, we simulated both attenuation and amplification interventions, then calculated the resulting changes in total depressive score and symptom prevalence. Nodes that produced the greatest absolute decreases in both sum scores and prevalence were identified as the most effective intervention targets.^50^

## Results

### Participant demographics

A total of 346 patients with BC met the inclusion criteria, with a mean age of 47.32 years (standard deviation [SD] ​= ​10.77 years). Additional information was provided in Table 1.

**Table 1:** Demographic characteristics of the sample (*N* ​= ​346).

Variable	Classification	*n* (%)
Age (years)		
	18–44	135 (39.0)
	45–59	177 (51.2)
	≥ 60	34 (9.8)
Marital status		
	Married	295 (85.3)
	Unmarried/Divorced/widowed	51 (14.7)
Education		
	Junior secondary and less	167 (48.3)
	High school/junior college	119 (34.4)
	Bachelor and more	60 (17.3)
Residence		
	Urban	159 (46.0)
	Rural	187 (54.0)
Medical insurance		
	Medical insurance for non-working urban residents	124 (35.8)
	Medical insurance for urban workers	177 (51.2)
	Other	45 (13.0)
Occupation		
	In-service	275 (79.5)
	No occupation	31 (8.9)
	Retirement	40 (11.6)
Household income (RMB)		
	< 3000	91 (26.3)
	3000–5999	126 (36.4)
	6000–9999	91 (26.3)
	≥ 10,000	38 (11)
Cancer stage		
	I	36 (10.4)
	II	143 (41.3)
	III	151 (43.6)
	IV	16 (4.7)
Fertility status		
	Born	319 (92.2)
	Not yet born	27 (7.8)
Family history of cancer		
	Yes	37 (10.7)
	No	309 (89.3)
Menopause or not		
	Yes	162 (46.8)
	No	184 (53.2)

### Network estimation for FCR and PC

**Fig. 1:**
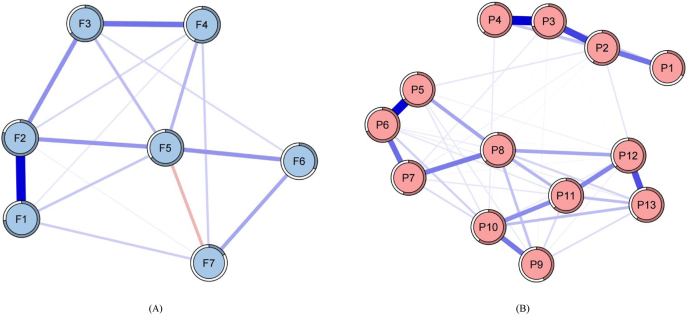
Network analysis visualization for fear of cancer recurrence (A) and pain catastrophizing (B).

The FCR network visualization was displayed in Fig. 1A. In this study, there were 23.81% (5/21) of the edges set to 0, and most of the edges were positive. However, a negative edge relationship is presented between F5 and F7. The strongest correlation was found between F1 and F2. Additionally, F2 (Str ​= ​1.285, Bet ​= ​8, Clo ​= ​0.030) was the core symptom in the network. There were 37.18% (29/78) of the edges set to 0 in the PC network. The network edges had an average weight of 0.049, with all PC symptoms showing positive associations with one another. The strongest correlation was found between P3 and P4. Additionally, P3 (Str ​= ​1.073, Bet ​= ​16, Clo ​= ​0.004) was the core symptom in the network. Fig. 1B showed the network analysis of PC. Supplementary Table S1 showed the components, component content, sample means, standard deviations, and network analysis of FCR and PC.

### Network estimation for the combination

**Fig. 2:**
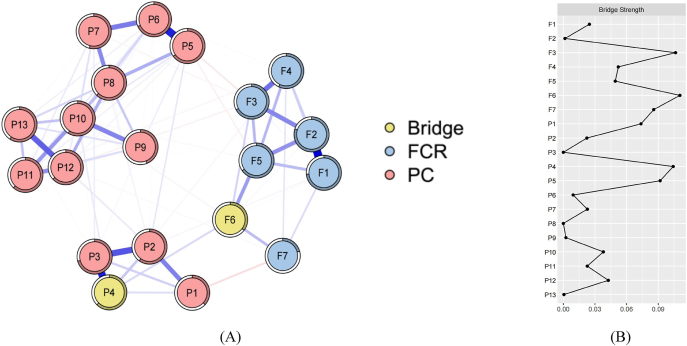
Network analysis visualization (A) and centrality metrics for the combination (B). FCR, fear of cancer recurrence; PC, pain catastrophizing.

Fig. 2A presented the estimated network for FCR and PC. In total, the complex network exhibited 106 non-zero edges (55.78%) out of a possible 190, and the strongest were F6–P4 (edge weight ​= ​0.085), F3–P5 (edge weight ​= ​−0.053), F5–P5 (edge weight ​= ​−0.037), and F1–P2 (edge weight ​= ​0.022). Specific details were shown in Table S2. The bridge strength was calculated for each node and was presented in Fig. 2B. From the bridge strength analysis, we identified F6 (bridge str ​= ​0.116) and P4 (bridge str ​= ​0.104) as the key bridges connecting the two communities. This indicated that F6 had the greatest impact on the PC symptom network and P4 contributed the most to the risk of the FCR symptom network. The 95% CI results for the edge weights indicated high accuracy. Furthermore, the CS coefficient in the network was 0.751 (cor ​= ​0.7), exceeding 0.5, which suggested sufficient stability. Full details were provided in Supplementary Fig. S1.

### Network comparison

Results from the NCT indicated that global expected influence did not differ significantly between early-stage and advanced-stage patients with BC (8.089 vs. 8.021, *S* ​= ​0.068, *P* ​= ​0.832). The results of Network Invariance Test did not show any difference in the network structure (*M* ​= ​0.199, *P* ​= ​0.437). Regarding Central Invariance Test, no significant differences were found for the comparison (All *P* ​> ​0.05). There was no significant difference in edge weights and the BEI among the two pairs of networks (All *P* ​> ​0.05). The comparative diagram of the specific network structure was shown in Supplementary Fig. S2.

### Bayesian network analysis

**Fig. 3:**
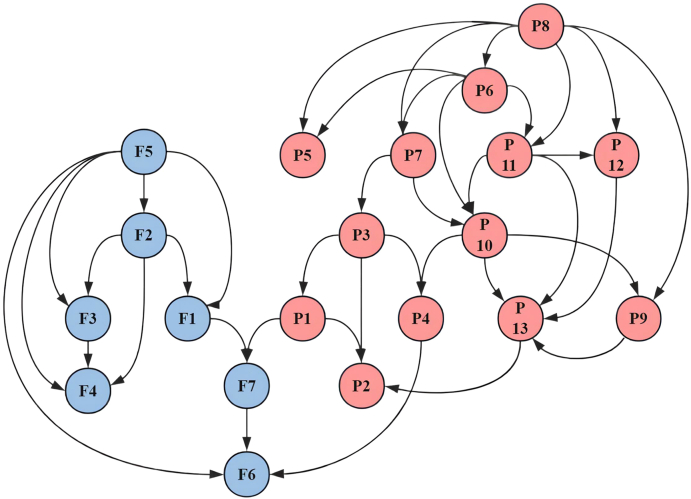
Bayesian network model for fear of cancer recurrence and pain catastrophizing.

In the Bayesian network (MBS ​= ​4.60; BF ​= ​1.80; BIC ​= ​−11002.56), 36 directed edges were preserved. The Bayesian network identified two parent nodes (Fig. 3). Firstly, the core path “F5→F2→F1→F7→F6” driven by F5 with a negative edge strengths (e.g., F5→F2 Str ​= ​−139.55) indicated a reduction in model BIC score when the edge is included, reflecting improved fit. Secondly, P8 indirectly affected F6 through the path “P8→P6→P7→P3→P4” (eg: P3→P4 Str ​= ​−138.75).

### Computer-simulated interventions

**Fig. 4:**
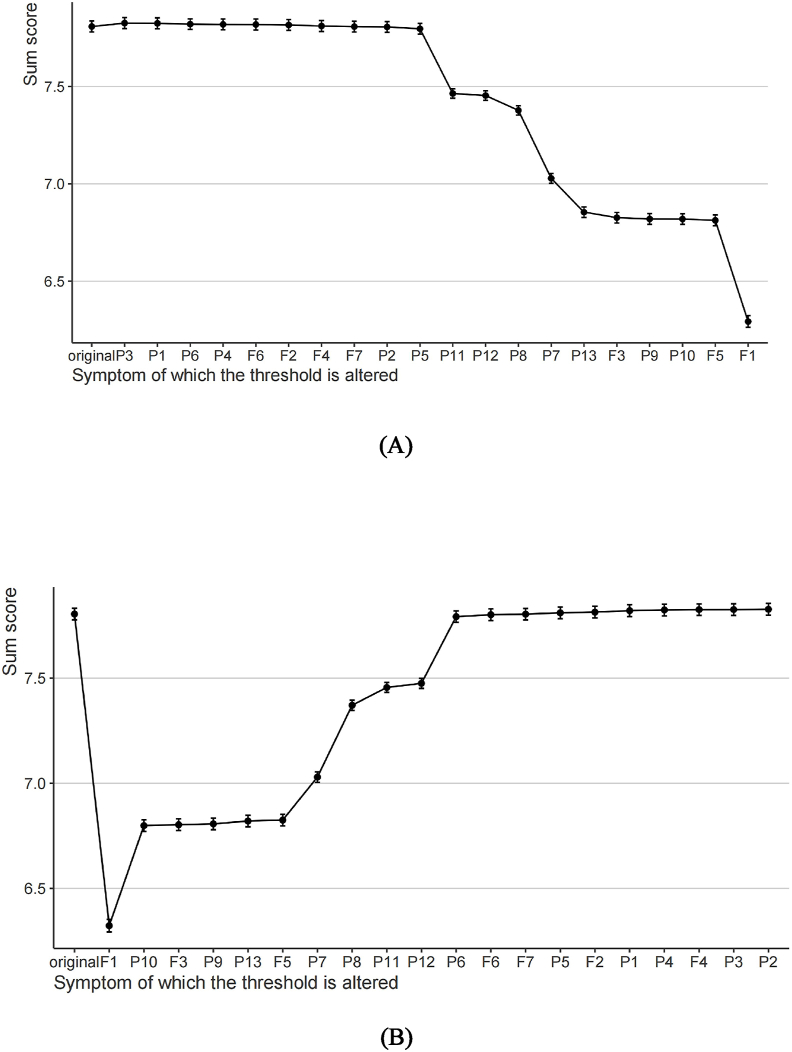
Computational simulation intervention for fear of cancer recurrence and pain catastrophizing: (A) Aggravating effect of symptom threshold alteration, (B) Alleviating effect of symptom threshold alteration.

Computer-simulated interventions showed that amplifying P3 led to the largest total score increase (ΔScore ​= ​+0.0174, 95% CI: 0.0079–0.0269, relative influence 18.2%), while attenuating F1 caused the greatest reduction (ΔScore ​= ​−1.4818, 95% CI: −1.5115 to −1.4521, relative influence −23.5%), with F1 attenuation also reducing symptom prevalence by 22.4% (95% CI: 19.7%–25.1%). All effects were validated via bootstrapping (10,000 samples, ≥ 75% consistency) and met clinical relevance thresholds (≥ 0.01 for aggravation, ≥ 1.0 for alleviation), identifying P3 and F1 as key intervention targets (Fig. 4). This suggests that P3 and F1 were the potential preventive targets for intervention.

## Discussion

While previous studies have applied network analysis to examine FCR or PC symptoms separately, this study uniquely combined these approaches by simultaneously examining FCR and PC symptom networks in BC patients, identifying bridge symptoms that connect these two networks, and investigating how tumor stage influences these bridge connections. This integrated network approach provides novel insights into the interconnected nature of psychological and physical symptom experiences.

### Main findings

This study identified a significant interaction among FCR symptoms, with most symptoms showing positive associations, but there was a negative side relationship between F5 (Insight) and F7 (Coping Strategies), indicating that these two nodes did occur simultaneously.^39^ F5 was also identified by Bayesian network analysis as a key node driving the symptom network, which confirmed hypotheses 1 and 3. The insight subscale assesses the degree of self-criticism related to FCR intensity.^40^ For example, some women with BC report access to disease-related information leading to a higher FCR.^19^ On the other hand, seeking support from family members is considered an important FCR coping strategy.^19^^,^^51^ In practice, cancer patients and their spouses frequently hide their worries and anxieties, avoiding open discussions about cancer-related information and emotions,^52^ resulting in failure to achieve the desired FCR coping effect. Thus, even though sometimes patients possess a high level of insight into FCR, they may instead increase the level of FCR after adopting personally negative coping strategies.

As in hypotheses 2 and 3, our study enhanced the understanding of the relationship between FCR symptoms and identified key intervention priorities. The strongest correlation was found between F1 (Triggers) and F2 (Severity), meaning the triggers of FCR and its severity had the greatest influence on each other. Besides, the results of computer intervention simulations also indicate that F1 is an effective target for regulating network behavior. Many studies^53^, ^54^, ^55^ have pointed out that individuals who perceive themselves to be at greater risk of cancer recurrence and are more susceptible to emotionally arousing stimuli from a variety of triggers show higher levels of FCR. Both these external and internal triggers activate cognitive responses associated with fear of recurrence,^54^ which in turn increases FCR, creating a vicious cycle. The network analysis also demonstrated that the core symptoms of FCR was F2. Thus, from a mechanistic perspective, the severity dimension emerged as the most central node within the FCR network among patients with BC. This finding appears logical, considering that severity directly represents the core concept of FCR itself, rather than merely associated factors.^41^^,^^56^ Practically, this could involve: (1) systematic assessment of patients' specific FCR triggers (e.g., medical appointments, physical symptoms, media exposure), (2) implementation of trigger management strategies such as cognitive restructuring techniques to reframe catastrophic interpretations of triggers, and (3) development of personalized coping plans for unavoidable triggers like follow-up appointments.

In this study, the strongest correlation was found between P3 (Terrible) and P4 (Awful), both items also belonging to the helplessness dimension. On the other hand, P3 was the most core node in the PC network, as well as a valid target in the results of computer intervention simulations. It is also worth noting that P8 (anxious) is a key parent node in the PC network. The Fear-Avoidance model could be utilized to elucidate the mechanisms underlying PC.^57^ Central to this model is the concept of fear towards pain. Patients with BC who have negative beliefs about their condition and pain may develop catastrophic thinking, which could lead to avoidant behaviors. These actions may exacerbate the fear of pain, triggering emotional distress (such as feeling terrible, anxious, or afraid) and physiological changes, thereby establishing a negative feedback loop.^58^ The assessment model proposes that feelings of helplessness may be linked to a secondary appraisal process, where individuals negatively assess their ability to cope with painful stimuli.^59^ In addition, the helplessness component of PC may provide a specific explanatory mechanism for the increased likelihood of comorbid depression among individuals suffering from more intense chronic pain.^60^ Clinically, these findings suggest that interventions targeting helplessness cognitions, particularly P3 (Terrible), through cognitive restructuring techniques may effectively disrupt the PC network. Additionally, integrating anxiety management strategies to address P8's parent role could prevent cascade effects throughout the network.

As mentioned in hypothesis 2, in our study, F6 (Reassurance) and P4 (It's awful and I feel that it overwhelms me) were the key bridging symptoms connecting different symptom clusters. Bridging symptoms may trigger and sustain comorbid psychiatric disorders.^61^ For reassurance-seeking behaviors, interventions should focus on building patients' tolerance for uncertainty rather than providing excessive reassurance, which may inadvertently reinforce the fear–pain cycle. For P4 (feeling overwhelmed by pain), interventions should address both the catastrophic appraisal and the sense of being overwhelmed. Clinicians can implement pain acceptance strategies combined with emotional regulation techniques to help patients manage overwhelming sensations without triggering FCR. The Cancer Threat Interpretation (CTI) model^62^ suggests that some survivors may experience pain as a conditioned fear stimulus, stemming from their previous understanding of pain's association with life-threatening illness. As a result, some survivors may become hypervigilant toward physical sensation of pain sensations as a marker of recurrence, resulting in heightened coexistence and cycling of PC and FCR.^19^ Compared to other patients, BC have more unmet needs,^63^ leading them to feel helpless and seek frequent reassurance. However, in analyses based on response theory (IRT), IRT identified several items with problematic item characteristic curves, particularly those in the Seeking comfort domain.^63^ Perhaps this is attributed to differences in cultural backgrounds.

In addition, this study found no statistical differences in the network structure when FCR and PC coexisted in patients with BC with different tumor stages, which again emphasizes the importance of psychological and other nonmedical factors associated with FCR.^64^ The probable explanation for this is that the diagnosis and treatment of the disease itself is a strong psychological stressor that could consistently evoke a generalized fear of disease progression and impending uncertainty, both in patients diagnosed with either early-stage or advanced BC.^15^

### Implications for nursing practice and research

Given that P3 (Terrible) was identified as the most central node within the PC network and yielded the largest simulated change, this symptom may be particularly amenable to cognitive restructuring techniques targeting maladaptive beliefs about pain^65^ like “This pain is unbearable” to “Pain can be managed.” Psychological education can normalize pain fluctuations, reducing their emotional impact. For “Triggers”, early screening tools can identify patients at risk of FCR escalation by assessing trigger sensitivity,^66^ enabling timely referral to CBT or psychoeducation. Prioritizing “Triggers” screening in advanced patients with BC can optimize resource allocation. For “Triggers” and “Reassurance”, by prioritizing these symptoms in clinical practice, health care providers could improve symptom management, reduce psychiatric comorbidity, and address unmet needs, ultimately enhancing the quality of life for BC survivors. Future research could explore individual variability and unique psycho-cognitive biases to inform the development of tailored supportive interventions. On the other hand, psychological symptom patterns may also be stable across disease stages, suggesting transdiagnostic or stage-independent symptom structures. The network comparison for subgroups may have lacked statistical power to detect subtle differences. In addition, the instruments used might not be sensitive enough to capture stage-specific variations.

### Limitations

This study examined the structure of fear of cancer recurrence and perceived pain in a sample of women with BC using network analysis. However, several limitations of the study should be acknowledged. First, this study focused exclusively on patients diagnosed with BC, so the findings may not generalize to survivors of other cancer types. Second, the study relied solely on self-reported psychometric data to assess psychological symptoms, without incorporating clinical diagnoses. Future studies could validate our findings by incorporating objective physiological indicators aligned with the same symptoms identified through professional clinical diagnosis. Additionally, the network structure in this study analyzed the relationships between variables at the population level. This suggests that the network structure may not manifest the same way in an individual case. Lastly, since the study included only patients with BC from southern China, caution should be exercised when generalizing the findings to populations in other regions of China or other countries.

## Conclusions

In summary, “Severity” and “Terrible” are the core symptoms of FCR and PC, respectively. The dimension “Assurance” and the item “Awful” act as bridging elements when FCR co-occurs with perceived pain experience in the network model presented here. The “Insight” in FCR and “Anxious” in PC as parent nodes, while simulations showed amplifying “Terrible” in PC maximized total score and connectivity, and attenuating “Triggers” in FCR minimized the score. Finally, no statistical differences in the network structure when FCR and PC coexisted in patients with early-stage versus locally advanced BC. Future studies could try to confirm the suggested intervention targets and explain the observed interconnectedness among symptoms.

## CRediT authorship contribution statement

**Furong Chen**: Data curation, Formal analysis, Methodology, Writing – original draft. **Yiguo Deng**: Investigation, Software. **Qihan Zhang**: Investigation, Software. **Siyu Li**: Investigation, Software. **Zhirui Xiao**: Data curation. **M. Tish Knobf** and **Zengjie Ye**: Supervision, Writing – review & editing. All authors have read and approved the final manuscript.

## Ethics statement

This study was approved by the Institutional Review Board (IRB) of The University of South China (Approval No. 2022NHHL013). All procedures were conducted in accordance with the ethical standards of the institutional research committee and the 1964 Helsinki Declaration and its later amendments. Written informed consent was obtained from all participants prior to enrollment in the study.

## Data availability statement

Data available on request due to privacy/ethical restrictions. The data that support the findings of this study are available on request from the corresponding author, ZY. The data are not publicly available due to their containing information that could compromise the privacy of research participants.

## Declaration of generative AI and AI-assisted technologies in the writing process

No AI tools/services were used during the preparation of this work.

## Funding

This research was funded by grants from the National Natural Science Foundation of China (Grant Nos. 72274043, 71904033), the Young Elite Scientists Sponsorship Program by CACM (Grant No. 2021-QNRC2-B08), Guangdong Philosophy and Social Science Foundation (Grant No. GD25YSH17), and Sanming Project of Medicine in Shenzhen (Grant No. SZZYSM202206014). The funders had no role in considering the study design or in the collection, analysis, interpretation of data, writing of the report, or decision to submit the article for publication.

## Declaration of competing interest

The authors declare no conflict of interest. Professor Zengjie Ye, the corresponding author, serves on the editorial board of the *Asia–Pacific Journal of Oncology Nursing*. The article underwent standard review procedures of the journal, with peer review conducted independently of Professor Ye and their research groups.
