# Supplementary material for: Fear of cancer recurrence and perceived pain in patients with breast cancer: A network analysis approach
Source: Asia Pac J Oncol Nurs. 2025 Jul 23;12:100763. doi: 10.1016/j.apjon.2025.100763 (PMC12355119; doi:10.1016/j.apjon.2025.100763)
Supplement: Multimedia component 1 [file mmc1.docx]

**Supplementary materials**

**Table S1. The components and network analysis of fear of cancer recurrence and perceived pain experience.**

| Components | Network analysis | | |
| --- | --- | --- | --- |
|  | Betweenness | Closeness | Strength |
| **Fear of cancer recurrence items** |  |  |  |
| F1: Triggers | 0 | 0.026 | 0.944 |
| F2: Severity | 8 | 0.030 | 1.285 |
| F3: Psychological Distress | 4 | 0.025 | 0.871 |
| F4: Functioning Impairment | 2 | 0.024 | 0.801 |
| F5: Insight | 10 | 0.033 | 1.178 |
| F6: Reassurance | 2 | 0.022 | 0.575 |
| F7: Coping Strategies | 0 | 0.021 | 0.678 |
| **Pain catastrophizing items** |  |  |  |
| P1: I worry all the time about whether the pain will end. | 0 | 0.004 | 0.467 |
| P2: I feel I can’t go on. | 44 | 0.005 | 0.941 |
| P3: It’s terrible and I think it’s never going to get any better. | 16 | 0.004 | 1.073 |
| P4: It’s awful and I feel that it overwhelms me. | 4 | 0.004 | 0.757 |
| P5: I feel I can’t stand it anymore. | 20 | 0.006 | 0.907 |
| P6: I become afraid that the pain will get worse. | 8 | 0.006 | 1.049 |
| P7: I keep thinking of other painful events. | 8 | 0.005 | 0.780 |
| P8: I anxiously want the pain to go away. | 22 | 0.006 | 1.069 |
| P9: I can’t seem to keep it out of my mind. | 2 | 0.005 | 0.643 |
| P10: I keep thinking about how much it hurts. | 18 | 0.006 | 0.975 |
| P11: I keep thinking about how badly I want the pain to stop. | 6 | 0.005 | 0.972 |
| P12: There’s nothing I can do to reduce the intensity of the pain. | 16 | 0.006 | 0.918 |
| P13: I wonder whether something serious may happen. | 22 | 0.006 | 0.887 |

**Table S2. Network estimation for the combination of fear of cancer recurrence and perceived pain experience.**

| Items | Network analysis of bridge | | |
| --- | --- | --- | --- |
|  | Strength | Betweenness | Closeness |
| F1 | 0.025 | 0 | 0.025 |
| F2 | 0.002 | 13 | 0.026 |
| F3 | 0.106 | 0 | 0.025 |
| F4 | 0.052 | 0 | 0.025 |
| F5 | 0.049 | 39 | 0.029 |
| F6 | 0.111 | 78 | 0.034 |
| F7 | 0.086 | 0 | 0.027 |
| P1 | 0.074 | 0 | 0.036 |
| P2 | 0.022 | 42 | 0.042 |
| P3 | 0.001 | 56 | 0.047 |
| P4 | 0.104 | 84 | 0.052 |
| P5 | 0.092 | 14 | 0.024 |
| P6 | 0.009 | 0 | 0.023 |
| P7 | 0.023 | 0 | 0.023 |
| P8 | 0.001 | 0 | 0.021 |
| P9 | 0.002 | 0 | 0.023 |
| P10 | 0.038 | 14 | 0.026 |
| P11 | 0.023 | 0 | 0.023 |
| P12 | 0.043 | 0 | 0.022 |
| P13 | 0.001 | 7 | 0.023 |
