# Supplementary figures and images for: Fear of cancer recurrence and perceived pain in patients with breast cancer: A network analysis approach
Source: Asia Pac J Oncol Nurs. 2025 Jul 23;12:100763. doi: 10.1016/j.apjon.2025.100763 (PMC12355119; doi:10.1016/j.apjon.2025.100763)

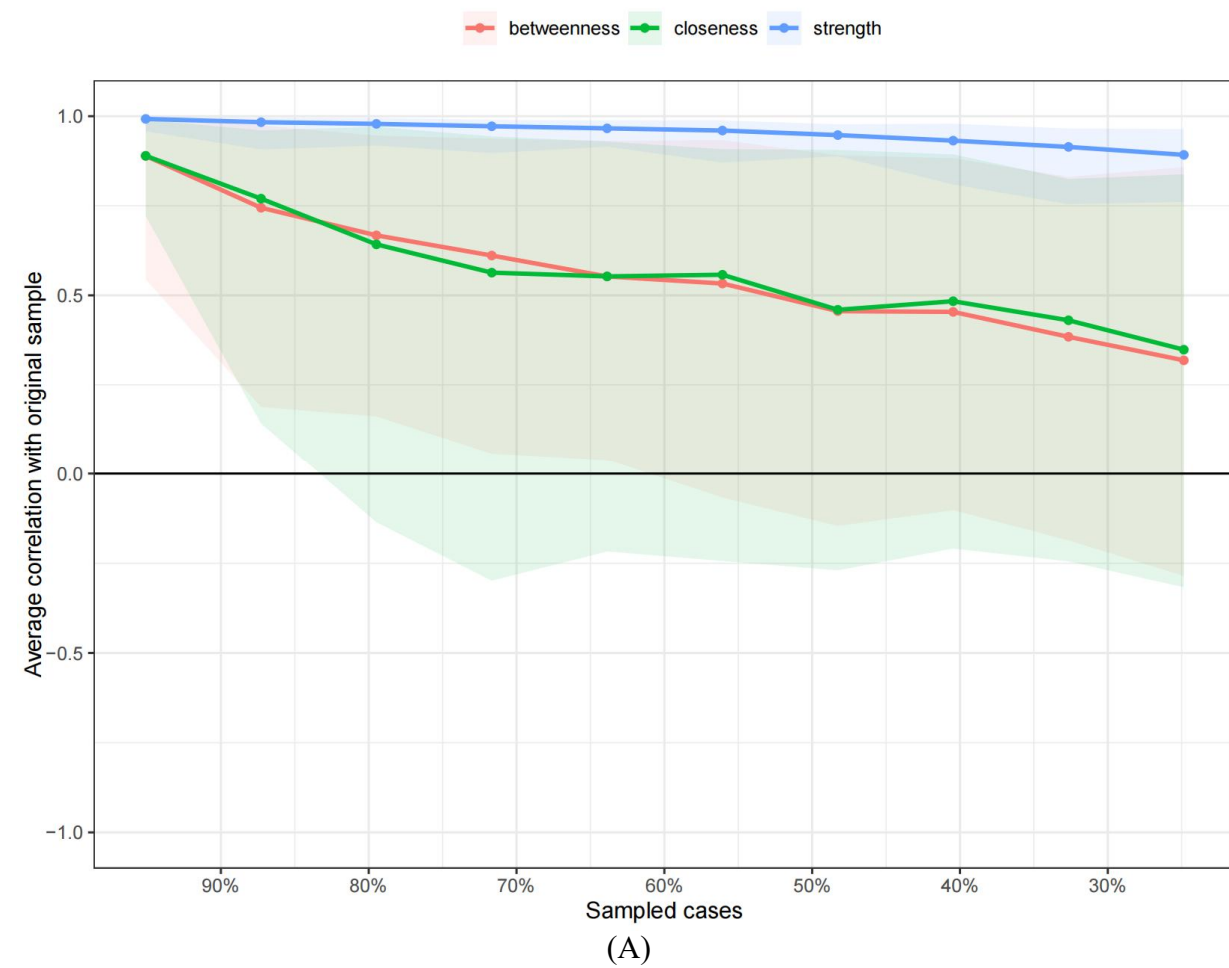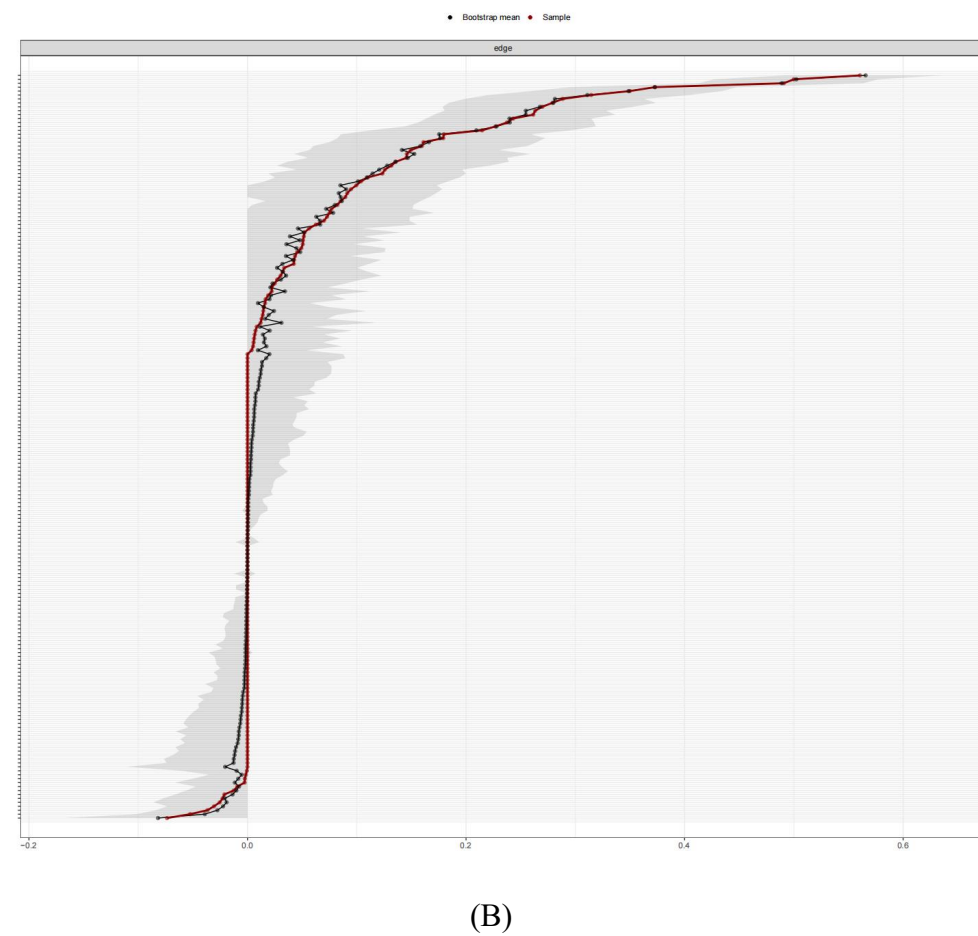

Supplement: Multimedia component 2 [file mmc2.pdf]

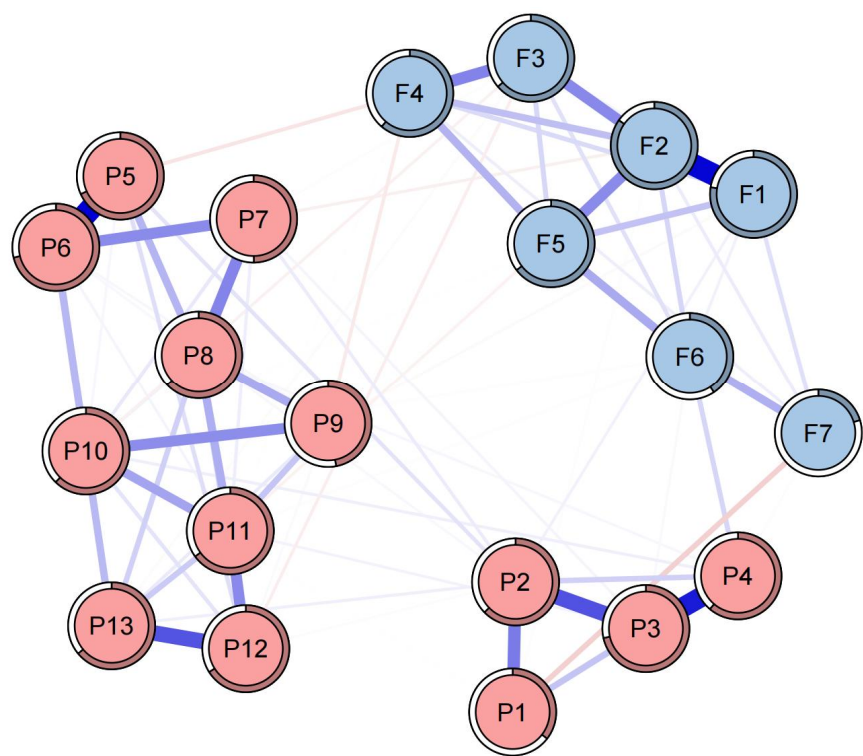

(A)

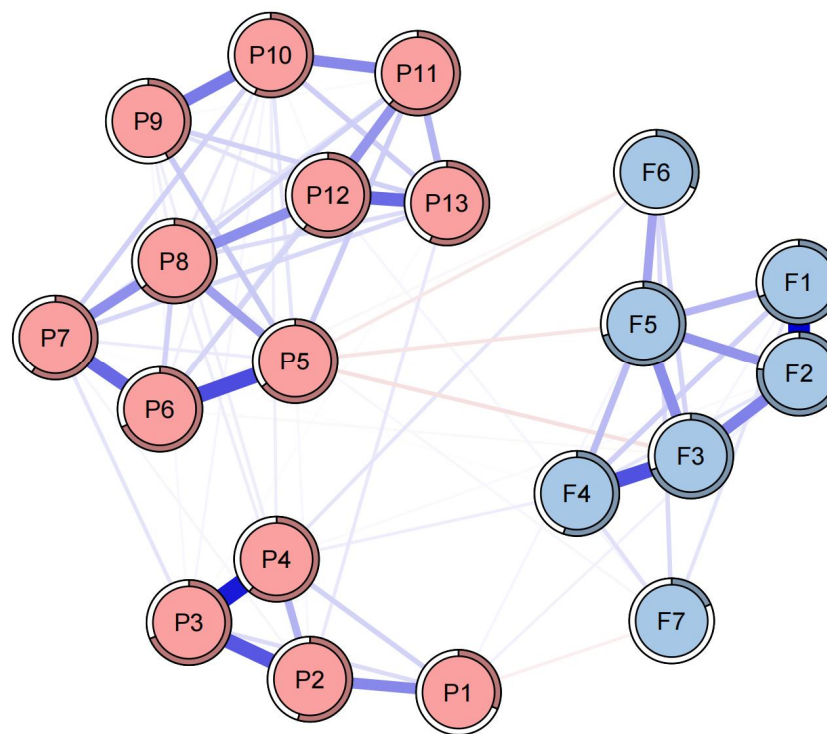

(B)

● FCR  
● PC

Supplement: Multimedia component 3 [file mmc3.pdf]
